# Association between community health center and rural health clinic presence and county-level hospitalization rates for ambulatory care sensitive conditions: an analysis across eight US states

**DOI:** 10.1186/1472-6963-9-134

**Published:** 2009-07-31

**Authors:** Janice C Probst, James N Laditka, Sarah B Laditka

**Affiliations:** 1 South Carolina Rural Health Research Center, Department of Health Services Policy and Management, Arnold School of Public Health, University of South Carolina 220 Stoneridge Drive, Suite 204, Columbia, SC 29210, USA; 2 Department of Public Health Sciences, University of North Carolina at Charlotte, 9201 University City Boulevard; Charlotte, NC 28223, USA

## Abstract

**Background:**

Federally qualified community health centers (CHCs) and rural health clinics (RHCs) are intended to provide access to care for vulnerable populations. While some research has explored the effects of CHCs on population health, little information exists regarding RHC effects. We sought to clarify the contribution that CHCs and RHCs may make to the accessibility of primary health care, as measured by county-level rates of hospitalization for ambulatory care sensitive (ACS) conditions.

**Methods:**

We conducted an ecologic analysis of the relationship between facility presence and county-level hospitalization rates, using 2002 discharge data from eight states within the US (579 counties). Counties were categorized by facility availability: CHC(s) only, RHC(s) only, both (CHC and RHC), and neither. US Agency for Healthcare Research and Quality definitions were used to identify ACS diagnoses. Discharge rates were based on the individual's county of residence and were obtained by dividing ACS hospitalizations by the relevant county population. We calculated ACS rates separately for children, working age adults, and older individuals, and for uninsured children and working age adults. To ensure stable rates, we excluded counties having fewer than 1,000 residents in the child or working age adult categories, or 500 residents among those 65 and older. Multivariate Poisson analysis was used to calculate adjusted rate ratios.

**Results:**

Among working age adults, rate ratio (RR) comparing ACS hospitalization rates for CHC-only counties to those of counties with neither facility was 0.86 (95% Confidence Interval, CI, 0.78–0.95). Among older adults, the rate ratio for CHC-only counties compared to counties with neither facility was 0.84 (CI 0.81–0.87); for counties with both CHC and RHC present, the RR was 0.88 (CI 0.84–0.92). No CHC/RHC effects were found for children. No effects were found on estimated hospitalization rates among uninsured populations.

**Conclusion:**

Our results suggest that CHCs and RHCs may play a useful role in providing access to primary health care. Their presence in a county may help to limit the county's rate of hospitalization for ACS diagnoses, particularly among older people.

## Background

### Rural Safety Net Providers

Access to primary health care in the US is affected by an individual's financial ability to pay for care, principally measured by insurance, and by the availability of a practitioner to provide services. Access in many rural counties is challenged at both the individual and the facility level: rural areas have proportionately more poor and uninsured persons than urban areas, and are served by fewer health care providers. [1,2] A number of urban counties are similarly at risk. [3] In both rural and urban settings, safety net facilities can have marked effects on population health. Two principal types of federally designated safety net facilities serve these areas: federally qualified community health centers (CHCs) and rural health clinics (RHCs). CHCs and RHCs are located in counties with demonstrated high need for care among at risk populations, and those that have been designated as rural, respectively.

Community health centers, administered by the Bureau of Primary Care, Health Resources and Services Administration (HRSA), have been the principal Federal vehicle for providing health care access to poor and uninsured persons. CHCs, which must be located in a medically underserved area, receive Federal grant funding that allows them to care for patients of limited financial means and to provide expanded services, such as transportation assistance, for vulnerable groups. Based on HRSA data, CHCs provided care for more than 15 million individuals in 2006, of whom nearly two thirds were of minority race/ethnicity. [4] Most CHC clients were at or below poverty (71%) and a substantial minority were uninsured (40%). [5] CHCs must accept all patients regardless of ability to pay, with a sliding-fee scale for the poor and uninsured. However, CHCs are expected to be "financially viable and cost-competitive;" thus, they are not required to provide free care to all patients. [6]

The Rural Health Clinic (RHC) program is directed toward the retention of physicians and other providers in rural areas. Established in 1977, it allows participating medical practices to receive higher reimbursement from Medicare and Medicaid, major payers for rural populations. [7] RHCs must be located in non-metropolitan Health Professional Shortage Areas (HPSAs), either a geographic shortage area (where the entire county lacks providers), or a population group shortage area (where specific types of individuals are underserved). Because the definition used for "rural" may either follow Federal guidelines or be set by a state governor, rural HPSAs can exist in counties that are classified as metropolitan or urban by the US Census. RHCs are not required to provide a full spectrum of primary care services; nor are they required to see all individuals seeking care regardless of need. As of 2005, 16 percent (590/3600) of RHCs stated that they would take all patients regardless of insurance status. [8] Although not required to accept uninsured individuals, RHCs actually derive a greater proportion of practice revenue from uninsured patients than do CHCs (15% versus 7%). [9] Advocacy groups, such as the National Rural Health Association, consider the RHC program a safety net function because of its role in rural physician recruitment and retention.

### Assessments of CHC and RHC effects on population health

We sought to clarify the contribution that CHCs and RHCs may make to the accessibility of primary health care, as measured by rates of hospitalization for ambulatory care sensitive (ACS) conditions. ACS conditions are those for which, in the consensus of medical experts, primary care of acceptable quality can reduce the frequency of hospitalization. [10-16]

### Ambulatory Care Sensitive Hospitalizations as a Measure of Access

ACS hospitalization as an indicator of primary care access assumes that quality outpatient care, by linking the patient to effective assessment, education, pharmacological management, and other treatment, reduces the likelihood that patients with specific diagnoses will need hospitalization. [10,15,16] While all relevant hospitalizations cannot be prevented, at the population level ACS hospitalizations have been found to be lower where other measures of access to care, such as provider availability, are higher. [17,18] Hospitalization rates for ACS conditions are higher in rural areas [19-22] and among nonwhites and individuals with low incomes. [23] ACS hospitalization rates are used by the Agency for Healthcare Research and Quality to measure access among minority populations, and in general assessments of safety net performance. [24]

### Evidence for CHC and RHC Effects on Population Health

Prior research has found that individuals insured by Medicaid who received most of their care at a CHC, compared with another single facility, were less likely to be hospitalized or to visit an emergency room for ACS conditions. [25,26] The presence of a CHC in a medical market area has been associated with lower ACS admission rates. [27] At the county level, the presence of a CHC has been shown to reduce ACS hospitalization rates among children. [28] Given this previous research, we anticipate that population-based hospitalization rates in counties served by CHCs will be lower than in counties lacking these facilities.

RHCs may also have effects on population health, although research in this area is sparse. Evidence suggests that an RHC can be financially beneficial to a sponsoring hospital. [29]. A person-level analysis, limited to Nebraska, found that hospitalizations in whole-county HPSAs containing an RHC were less likely to involve an ACS condition than those in counties without an RHC. [30] This study, which did not examine population-level risks for ACS hospitalization, is the only previous research examining the association between the presence of an RHC and population health.

Our analysis expands on previous work by examining effects of CHCs, RHCs, and both facilities in combination, on population level access to care, as measured by county-level hospitalization rates for ACS conditions. Our primary analysis examines these rates in the general population, stratified by age. Because CHCs have a specific mission to help medically indigent populations, including uninsured persons, we also examine the association between the presence of CHCs and/or RHCs and ACS admission rates among the uninsured.

## Methods

### Sample

We used a cross-sectional, ecologic design to explore relationships between county-level rates of ACS diagnoses and county level covariates, including the presence of a CHC, RHC, or both. ACS hospitalization rates are calculated based on hospitalizations of persons who reside in the county, regardless of where the hospitalization takes place. The unit of analysis is an age- and county-specific rate, calculated based on discharges of county residents. Counties were used, rather than smaller geographic units, because most of the data elements needed for multivariate analysis are available only at this level. The study was approved by the Institutional Review Board of the University of South Carolina.

Data were drawn from the 2002 State Inpatient Databases (SIDs). The SIDs, compiled at the state level and supported for research use by the Agency for Healthcare Research and Quality, contain discharge records for all hospitalizations in participating states (100 percent). Only fifteen states included information about patients' counties of residence in 2002. Budgetary constraints coupled with the per-state cost for SID files limited the analysis to eight states: Colorado, Florida, Kentucky, Michigan, New York, North Carolina, South Carolina, and Washington. These states were chosen to provide at least one state in each of the four major Census Divisions of the US, and to offer a large number of counties with CHCs, RHCs, or both facilities. The presence of a CHC or RHC in each county was determined using Area Resource File data for the year 2002. Four mutually exclusive categories were created: CHC but no RHC, RHC but no CHC, both (CHC and RHC), and neither facility. Across all counties, 59 (10.2%) had a CHC and not an RHC; 139 (24.0%) had an RHC but not a CHC; 27 had both facilities (4.7%), and 354 (61.1%) counties had neither facility. Counties were distributed by state as follows: Colorado, 63 counties (10.9%); Florida, 66 counties (11.4%); Kentucky, 120 counties (20.7%); Michigan, 83 counties (14.3%), New York, 62 counties (10.7%); North Carolina, 100 counties, 17.3%; South Carolina, 46 counties (7.9%), and Washington, 39 counties (6.7%).

### Measurement of ACS Conditions

We used definitions for ACS diagnoses from the Agency for Healthcare Research and Quality. [31] The ACS conditions for adults are specific diagnoses for asthma, angina (without procedure), congestive heart failure (CHF), bacterial pneumonia, chronic obstructive pulmonary disease (COPD), dehydration, diabetes long-term complications, diabetes short-term complications, hypertension, lower-extremity amputation for individuals with diabetes, perforated appendix, uncontrolled diabetes, and urinary tract infection. For children, the ACS conditions include asthma, bacterial pneumonia, dehydration, perforated appendix, gastroenteritis, and urinary tract infection. The precise definitions used in this research account for a variety of exclusions detailed in technical specifications that are readily available from the AHRQ [31]. For children, for example, hospitalizations for asthma are excluded if there is evidence of cystic fibrosis or anomalies of the respiratory system. A hospitalization for any of these diagnosis is considered to be a hospitalization for an ACS condition. We did not attempt to study hospitalizations for individual diagnoses because of the instability of rates in counties with very small populations, and also because most research in this area uses the combined indicator.

### Analytic approach

We examined adjusted rates of ACS hospitalization in counties with a CHC, an RHC, or both, and compared these to the analogous rates in counties with neither facility. We calculated ACS rates separately for children (0–17), working age adults (18–64), and older individuals (65 and over). To ensure stable rate estimation, we established population-based criteria for county inclusion before conducting data analysis. County-level population estimates for 2002 were drawn from the 2005 Area Resource File (n = 579 counties). We included a county in the rate analysis for children and for working age adults only if it had at least 1,000 persons ages 0 – 17 (children) or ages 18 – 64 (working age adults). For age 65 and over, we included a county only if it had at least 500 persons in that age group; the threshold of inclusion was lower for older persons because of their higher ACS admission rates. These criteria excluded 21/579 counties from the analysis for children (3.6%), 5/579 counties from the analysis for working age adults (0.9%), and 12/579 counties from the analysis for older adults (2.1%). We considered an alternative approach, retaining all counties in the analysis and adjusting standard errors to account for heteroskedasticity. We judged that this approach might not adequately account for unrepresentative high or low ACSH rates that could appear among such small populations. Such unrepresentative rates could introduce bias into the estimations, because they could be attributable to even small random variations in the number of individuals hospitalized for ACSCs in these small populations, rather than to differences in access to primary health care. A comparison of mean county population and mean number of ACS discharges for included and excluded counties is provided in Table 1.

**Table 1:** Mean Number of Persons in Each Age Range for Included and Excluded Counties, and Mean Number of ACSC Hospitalizations in Each Age Range in these Counties

	**Ages 0–17**		
	
	Number of Counties	Mean Population	Mean Number of ACSC Discharges
Included Counties	559	31,535	152.4

Excluded Counties	20	614	1.8

County Total	579		

	**Ages 18–64**		

Included Counties	574	78,778	687.1

Excluded Counties	5	661	4.6

County Total	579		

	**Ages 65+**		

Included Counties	Number of Counties	Mean Population	Mean Number of ACSC Discharges

Excluded Counties	567	16,912	1,111

County Total	12	276	14.7

	579		

As noted in the introduction, CHCs and RHCs are located only in specific county types and are not randomly distributed across the US. As illustrated in Table 2, counties with CHCs and/or RHCs differ from counties in those same states with neither facility in several characteristics, including HMO penetration and proportion of the population that is uninsured. To adjust for differences between studied counties and counties with neither facility, adjusted analyses controlled for the county characteristics listed in Table 2.

**Table 2:** Characteristics of counties, by CHCs/RHCs in the county, studied states, 2002.

	Counties in studied states only, with:						All U.S. Counties
	
SID Sample, n = 579	CHC Only		RHC Only	Both CHC and RHC		Neither facility	
Number of Counties:	59		139	27		354	3,168

**Resources in county:**							

MD/DO per 10,000 population	12.9		10.3	14.3		12.3	12.1

Beds per 10,000 population	3.6		3.2	3.9		3.2	3.9

Number of hospitals with emergency department	1.7		1.0	1.4		1.6	1.3

HMO penetration rate	25.3	***	9.4	10.6		14.2	11.4

ED visits per 1,000 population	337		372	382		330	351

Non-metropolitan county (%)	23.7	***	79.9	66.7	***	58.8	65.3

**Characteristics of county population:**							

Percent of population that is:							

African American	20.3		17.5	16.2		16.4	9.5

Hispanic white	6.4		6.6	7.7		6.4	5.3

Asian	1.7		1.6	3.5		2.1	1.0

American Indian/Native American	2.1		5.1	2.1		2.3	1.9

Population change, 1990 – 2000 (%)	10.4		12.5	8.5		13.0	8.1

Percent of population with less than a high school education	24.9		24.3	24.0		25.0	22.6

Population per square mile	167		141	183		219	23

Percent of population that is unemployed	7.3		6.8	6.3		7.0	7.1

Percent uninsured, aged 18–64	19.0		20.8	22.1	**	18.9	19.6

Percent uninsured, age 17 or less	12.0		12.9	13.3	*	11.4	12.4

Median household income	35,595		35,179	36,835		35,844	35,363

**Death rate per 10,000 due to:**							

Cardiovascular disease	18.3		15.6	17.1		17.0	20.7

Chronic obstructive pulmonary disease	5.1		4.6	5.1		4.7	5.1

Diabetes	2.6		2.2		2.2	2.2	2.8

Liver disease	1.0		0.9		0.9	0.9	0.9

Data Source: Authors' analysis using year 2002 State Inpatient Databases (8 states), and the 2002 Area Resource File (ARF). Note that ARF death rates are based on 3-year average. Statistical tests compare the indicated category to counties that have neither facility type; statistical tests are t-tests, except for the chi-square test for non-metropolitan counties (conducted as a single chi-square for all CHC/RHC combinations). **Note: **3168 is the number of US counties in states. Excludes ARF counties from U.S. Territories (Guam, Puerto Rico, etc.).

***p < .001; **p < .01; *p < .05

The models for this study are based on Andersen's (1995) conceptualization of use of health services as resulting from the multiple influences of the external community and health services environment, population characteristics, health behavior, and outcomes. [32] Variables representing health system characteristics and use included physician supply, bed supply, number of hospitals with an emergency department, emergency department visit rates, and managed care penetration rates. Physician supply is generally inversely related to ACS hospitalization rates [17,20,33], but a positive relationship [34] and no relationship [35,36] have also been found. Managed care penetration has been found to be inversely related to ACS hospitalization rates. [37,38] County characteristics measured included racial/ethnic composition of the population (proportions that are non-Hispanic black, Hispanic, Asian American, and American Indian/Native American), population change 1990 – 2000; the percent of the population with less than a high school education, the unemployment rate, population per square mile, and whether the county was classified as metropolitan (urban) or non-metropolitan (rural). [39] The racial/ethnic composition of the population is included to adjust for differing patterns of health and health care use among minorities. [17,40-42] Population change, education levels, and unemployment are used as measures of the financial and economic status of the county as a whole. Population density is used, in addition to rural status, to adjust for differences within rural counties. Including a rural/urban variable in the model does not introduce unacceptable colinearity with the covariate representing RHCs, because a notable proportion of counties with RHCs are classified as metropolitan (Table 2). Resource characteristics included median household income and the percent of the population estimated to lack health insurance. Estimates of the uninsured population in each county were obtained from the U.S. Census. [43] Consistent with previous research, we included four covariates to control for county health burdens: unadjusted death rates from cardiovascular disease, chronic obstructive pulmonary disease, diabetes, and liver disease. [32] Table 2 provides a full description of these parameters across county types. With the exception of county-level estimates of the uninsured population, all variables are drawn from the Area Resource File.

Multivariate Poisson analysis was used to calculate adjusted rate ratios comparing counties with one or more CHCs, one or more RHCs, or at least one CHC plus at least one RHC, to counties having none of these facility types, while holding other county characteristics equal. The rate ratio is the ratio of the mean value of ACS hospital admission rates across counties of a given type, separately estimated for each age group, where the mean rate for a county type of interest (such as counties with both a CHC and an RHC) is the numerator. The denominator is the corresponding rate for counties having neither a CHC nor an RHC, the reference category. The rate ratio is obtained by exponentiating the estimate of interest from the Poisson analysis. Rate ratios less than 1.00 suggest that the hospitalization rate in the county type of interest was lower than the rate in the reference category.

For calculating rates among uninsured adults, we used Census estimates of the number of uninsured adults in each county as the denominator. We made the assumption that nearly all such persons are younger than 65, as most older people are covered by Medicare. For the separate analysis of children, the denominator was the Census estimate of uninsured children. The numerator specific to each age group in each county was the number of ACS admissions for which the payment source was identified as "self pay" in the discharge record. This value may not precisely equal the uninsured population, as some self-pay admissions may later have been converted to an insurer; however, it is reasonable to assume that the number of cases in which this occurred is relatively small. Measurement errors, if present, might have the greatest effect on ACS admission rates among children, which are generally quite low and thus could be affected by small changes.

## Results

### ACS Hospitalization Rates across County Populations

Unadjusted ACS hospitalization rates were lowest among children and markedly higher in the 65 or older population (Additional File 1). Unadjusted ACS rates among children did not differ by CHC/RHC availability. ACS hospitalization rates in the working age and age 65 or above populations were significantly lower in counties with a CHC than in counties with neither facility; rates in counties with an RHC only, or both facilities, did not differ from those in counties with neither facility.

In adjusted analysis, the presence of a CHC or RHC in the county was associated with ACS hospitalization rates for children only for the comparison of counties with both a CHC and RHC with those having neither facility. The rate ratio comparing these counties, 1.30 (95% Confidence Interval, CI 1.10–1.55), suggests that ACS hospitalizations are more common in counties with both facility types (Additional files 1 and 2). Among working age adults, the ACS hospitalization rate in counties having a CHC was 0.86 of the rate in counties with neither facility type (95% CI 0.78–0.95). ACS hospitalization rates in counties with an RHC only, or with both facility types, did not differ from those in the comparison group. Among older adults, counties with either safety net facility had lower ACS hospitalization rates than counties with none. The rate in counties with one or more CHCs, but no RHC, was 16% lower than those with neither facility type (rate ratio, RR, 0.84, CI 0.81–0.87). The rate in counties with one or more RHCs, but no CHC, was 4% lower than that in counties with neither facility type (RR 0.96, CI 0.94–0.99). The rate in counties with at least one CHC and at least one RHC was 12% lower than in those with neither facility type (RR 0.88, CI 0.84–0.92).

We examined the residuals from these analyses to identify whether some states or county clusters might systematically exhibit an association between CHCs/RHCs in the opposite direction from these estimated results. Although ACS hospitalization rates were generally higher in Kentucky than in the other states in the analysis, with greater variation in these rates in Kentucky as well, there was no indication that the rates might systematically depart in direction from these adjusted averages for particular states or clusters of counties.

Multiple county characteristics in addition to CHC/RHC presence were associated with ACS hospitalization rates, particularly among older adults (Additional File 2). Factors with similar effects across all age groups included HMO penetration (lower rates), positive population change (lower rates), and cardiovascular disease death rates (greater rates). Residence in a non-metropolitan county was associated with notably lower ACS hospitalization rates among children, and modestly lower rates among older adults, with facility availability held constant.

### ACS Hospitalization Rates among Estimated Uninsured County Populations

There was no evidence that the presence of a CHC or RHC was associated with lower ACS hospitalization rates for uninsured children (Tables 3 and 4). In unadjusted results for uninsured working age adults, counties with at least one CHC, but no RHC, had an average ACS hospitalization rate per 1,000 uninsured persons of 8.44, compared with an average rate of 10.40 for counties with neither safety net facility (p = 0.0029). When demographic and health resource characteristics of the counties were controlled in multivariable analysis, there were no differences in the rates of ACS hospitalization among uninsured persons associated with the presence of safety net facilities in the county.

**Table 3:** County-level ACS Hospitalization Rates among Estimated Uninsured Populations, by Age Group, Eight States, 2002.

	Unadjusted Rate per 1000	95% confidence interval	P-value
Children (Ages 0 – 17)			

CHC Only (n = 27)	1.65	(0.98, 2.31)	0.4213

RHC Only (n = 50)	1.12	(0.71, 1.53)	0.3267

RHC&CHC (n = 12)	2.40	(-0.19, 4.99)	0.3973

Neither (n = 160)	1.36	(1.06, 1.47)	n/a

Working age adults (Ages 18 – 64)			

CHC Only (n = 59)	8.44	(7.42, 9.46)	0.0029

RHC Only (n = 137)	11.18	(10.18, 12.17)	0.2261

RHC&CHC (n = 27)	13.20	(9.40, 17.00)	0.1499

Neither (n = 308)	10.40	(9.62, 11.18)	n/a

**Source: **Authors' analysis using year 2002 State Inpatient Databases representing 8 states, and the 2002 Area Resource File; analysis for children limited to counties having at least 1,000 uninsured children ages 0–17; analysis of adults limited to counties having at least 1,000 uninsured adults ages 18–64.

**Table 4:** Factors influencing county-level hospital ACS hospitalization rates among estimated uninsured populations, eight states, 2002

	Children (ages 0 – 17) 249 counties			Working age adults (ages 18 – 64) 571 counties		
	Model Coefficient	SE	p-value	Model Coefficient	SE	p-value

**Facilities (ref: neither)**						

CHC Only	-0.0778	0.2048	0.7042	-0.0089	0.0546	0.8702

RHC Only	-0.1159	0.1606	0.4706	-0.0315	0.0321	0.3266

RHC&CHC	0.4973	0.2125	0.0193	0.0702	0.0579	0.2255

**Resources in county:**						

MD/DO per 10,000 population	-0.0002	0.0062	0.9760	0.0001	0.0018	0.9340

Beds per 1,000 population	0.0106	0.0265	0.6878	0.0122	0.0045	0.0070

Number of hospitals with Emergency Dept.	0.0130	0.0384	0.7355	0.0227	0.0094	0.0154

HMO penetration rate	-0.0088	0.0045	0.0518	-0.0014	0.0013	0.2761

ED visits per 100	0.0040	0.0028	0.1605	0.0036	0.0005	< .0001

Non-metropolitan county (v metro)	-0.2756	0.1597	0.0843	-0.0549	0.0405	0.1761

**Characteristics of county population**						

Percent population that is:						

African American	-0.0043	0.0048	0.3724	0.0019	0.0011	0.0840

Hispanic white	-0.0456	0.0280	0.1036	-0.0101	0.0025	< .0001

Asian	0.0710	0.0418	0.0898	-0.0096	0.0161	0.5527

American Indian/Native American	0.2141	0.0390	< .0001	-0.0056	0.0045	0.2193

Population change, 1990 – 2000, %	-0.0216	0.0072	0.0025	-0.0017	0.0014	0.2269

Percent with less than high school education	0.0073	0.0104	0.4787	0.0401	0.0023	< .0001

Population per square mile (/100)	0.0043	0.0013	0.0011	-0.0002	0.0006	0.6908

Percent unemployed (/10)	-0.7131	0.2180	0.0011	0.0884	0.0410	0.0310

Percent uninsured*	-0.0147	0.0139	0.2902	0.0006	0.0052	0.9095

Median household income (thousands)	-0.0241	0.0139	0.0819	-0.0011	0.0013	0.4113

**Death rates (×10,000) for:**						

Cardiovascular disease	0.0031	0.0093	0.7412	0.0124	0.0023	< .0001

Chronic obstructive pulmonary disease	0.0890	0.0444	0.0447	0.0090	0.0090	0.3194

Diabetes	-0.1353	0.0584	0.0206	0.0194	0.0126	0.1226

Liver disease	-0.3647	0.1294	0.0048	0.0620	0.0230	0.0070

* Estimated percent uninsured among children (ages 0–17) in the model for children, among working age adults (ages 18–64) for the model for adults.

## Discussion

Our findings confirm and extend previous research suggesting that CHC presence may be associated with improved access to care, or receipt of care, for certain age groups [25-28]. At the population level, the presence of a CHC in a county was associated with lower ACS admission rates among both working age and older adult populations, when compared to counties that had neither a CHC nor an RHC available. The presence of an RHC in the county was not associated with lower ACS hospitalization rates among children or working age adults. This conflicts with the single previous study exploring RHC effects. However, the work by Zhang and colleagues [30] was restricted to a single type of county (HPSA) in a single state (Nebraska), and was also limited to estimating relative risks of having an ACS diagnosis versus other diagnosis among hospitalized individuals. Their findings may thus be geographically and structurally restricted, and may not reflect risks of ACS hospitalization at the population level across a more diverse region.

### Possible Associations among Older Individuals

Among older adults in the present study, ACS hospitalization rates were lower in counties with CHCs or RHCs, alone or together, compared with counties having neither facility. These rate differences provide suggestive evidence that CHC and RHC location in a county may be associated with greater accessibility or quality of primary health care.

Adjusted admission rates for ACS conditions among older adults were 12% lower among counties that had a CHC plus an RHC, compared with those having no safety net facility, and were 16% lower across counties having only a CHC. The association between CHC presence and lower ACS admission rates may be a function of CHC availability, paralleling earlier research [33], or may be related to chronic disease management programs in CHCs [44]. Further research linking older adults to specific safety net facilities is needed to clarify the findings of the present ecological analysis. The reduction in ACS hospitalization rates among older adults in counties with RHCs was small, but consistent with previous research linking CHC/RHC availability to reduced hospitalization among Medicare beneficiaries. [21]

It is possible that lower ACS hospitalization rates for older individuals in counties having a CHC or RHC are an artifact of differences between counties with and without such facilities, even after adjusting for the factors noted in Table 2. For example, counties with CHCs have markedly higher HMO penetration rates than other counties; higher HMO penetration is associated with lower hospitalization rates. [37]

### Minimum associations among children

The presence of a CHC or RHC in the county of residence was associated with ACS hospitalization rates only in counties having both of these facilities, where it was associated with higher rates. These findings contradict previous research suggesting that CHC presence reduced ACS admission rates among children [28]. The study by Garg and associates [28], however, was restricted to a single state (South Carolina) and may not be typical of other U.S. states. The findings of no association between CHC or RHC presence and hospitalization rates are consistent with other research, restricted to urban counties, finding no CHC effects on pediatric hospitalization when physician supply was held constant. [33] The association of CHC plus RHC presence with higher hospitalization rates is unexpected. Further research is needed to ascertain whether this finding was an artifact of the small number of counties studied, or is more generally relevant.

### Absence of effects among the uninsured

While the presence of a CHC in a county was associated with lower ACS hospitalization rates at the population level, it did not have parallel associations for the estimated uninsured population. Similarly, the presence of an RHC in a county was not associated with lower estimated hospitalization rates among the uninsured. As noted earlier, RHCs are not required to accept uninsured individuals, and a minority of RHCs report doing so. [8] Thus, it would not be anticipated, on the basis of mission, that RHCs would improve access to care for the uninsured. However, expansion of the number of CHC access points across the nation has been a key element of the Federal approach to the uninsured population since 2002. [45] Absence of CHC effects for the uninsured, assuming that the present ecological study is confirmed by additional research, could indicate the need for a revised approach to improving access.

Further research is needed to clarify individual and institutional barriers to the provision of quality primary care to uninsured populations. Analysts have suggested that CHC expansion has not been sufficient to keep pace with the increasing number of uninsured persons caused by the steady erosion in private insurance. [45] Further, since minorities are more likely than whites to lack insurance, addressing the problem of disparities among the uninsured is key to addressing racial/ethnic disparities in general. [45] Finding measures that will counteract any barriers experienced by uninsured populations will thus contribute to the reduction of race based, as well as insurance based, differences in care.

The present study had several methodological limitations. First, like most studies using the ACS indicator [e.g., [23,33,36]], the analysis was ecological. While the county of residence of hospitalized persons was identified in the SID, no information was available regarding ambulatory care, beyond physician supply and the presence of the types of facility studied. Thus, we are unable to state what proportion of persons in a county received their care from a CHC or RHC, and thus could not directly address the role of these institutions in limiting ACS admissions. Second, the analysis is based on a convenience sample of states providing patient residence data in 2002. An analysis using more recent data for the same states might yield different results, given the expansion in CHC treatment sites and increasing adoption of Health Disparities Collaborative activities in recent years. [44] In addition, the number of states providing residence data to the SIDs has increased; an analysis based on all available states might have different findings. On the other hand, the results of the present analysis are applicable to the large population of the eight states we studied, totaling 72.3 million. Third, the study did not control for the potential presence of additional safety net facilities, such as free clinics. Such facilities could be more likely to locate in counties served by CHCs or RHCs, enhancing the effect of the latter; alternatively, they could be located in other counties and reduce the comparison to study counties. Fourth, the study used estimates of the number of uninsured persons in each county as the denominator for calculating hospitalization rates among the uninsured. While Census estimates offer reasonably accurate estimates of the uninsured population, our results may be limited by measurement error in these estimates. Conversely, insurance information provided in the discharge summary (from which the numerator was calculated) may be inaccurate if information about eventual payor was added at a later point. Fifth, the data did not permit the identification of individuals, and therefore of repeated hospitalizations for the same individual. Repeated hospitalizations for the same individuals may bias the estimated results. Given that the study period was limited to a single year, this factor is unlikely to have affected the results notably. However, it is possible that individuals with ACS hospitalizations are at higher risk of early re-hospitalization due to inadequate follow-up after discharge, given that ACS hospitalizations suggest a problem with the accessibility or quality of primary health care. Sixth, several control variables were obtained from the Area Resource File (ARF), a data source that is commonly used by health services researchers for county-level measures. Supported by the Health Resources and Services Administration, the ARF provides measures for many health-related variables for all U.S. counties. However, some of its measures, such as those from the American Hospital Association, are subject to survey error.

## Conclusion

Our results suggest that CHCs and RHCs may play a useful role in providing access to primary health care. Their presence in a county is associated with a lower rate of hospitalization for ambulatory care sensitive conditions among older adults, and in some circumstances for working age adults. Further research is needed to verify to potential relationships suggested by this study, and to understand the role of CHCs and RHCs in access to health care for children.

## Competing interests

The authors declare that they have no competing interests.

## Authors' contributions

JCP developed the study concept, identified applicable data, and drafted the manuscript. JNL designed and implemented the statistical and modeling approach, and helped draft the manuscript. SBL helped to develop the study design and modeling approach, and helped draft the manuscript. All authors read and approved the final manuscript.

## Pre-publication history

The pre-publication history for this paper can be accessed here:



## Supplementary Material

Additional file 1 **Unadjusted Admission Rates and Adjusted Rate Ratios for ACS Hospitalizations, Eight States, 2002**. Click here for file

Additional file 2 **Factors influencing county population-level ACS hospitalization rates, by age group, eight states, 2002**. Click here for file
