# Supplementary material for: Association between community health center and rural health clinic presence and county-level hospitalization rates for ambulatory care sensitive conditions: an analysis across eight US states
Source: BMC Health Serv Res. 2009 Jul 31;9:134. doi: 10.1186/1472-6963-9-134 (PMC2727502; doi:10.1186/1472-6963-9-134)
Supplement: Additional file 1 — Unadjusted Admission Rates and Adjusted Rate Ratios for ACS Hospitalizations, Eight States, 2002. [file 1472-6963-9-134-S1.doc]

Additional File 1. Unadjusted Admission Rates and Adjusted Rate Ratios for ACS Hospitalizations, Eight States, 2002.

|  | Number of ACS Discharges  (mean across counties) | Unadjusted Rate per 1,000 | 95% confidence interval | P-value | Adjusted  Rate Ratio | 95% confidence interval | P-value |
| --- | --- | --- | --- | --- | --- | --- | --- |
|  | Children (Ages 0 – 17) | | | | | | |
| CHC Only (n=58) | 653.2 | 4.62 | (4.06, 5.18) | 0.3063 | 1.07 | (0.94, 1.22) | 0.3234 |
| RHC Only (n=138) | 68.0 | 4.98 | (4.28, 5.69) | 0.9474 | 1.07 | (0.98, 1.18) | 0.1336 |
| RHC&CHC (n=27) | 160.6 | 5.56 | (3.98, 7.15) | 0.5013 | 1.30 | (1.10-1.55) | 0.0025 |
| Neither (n=335) | 99.0 | 5.01 | (4.51, 5.51) |  | referent | referent |  |
|  | Working age adults (Ages 18 – 64) | | | | | | |
| CHC Only (n=59) | 2666.7 | 8.44 | (7.42, 9.46) | 0.0036 | 0.86 | (0.78, 0.95) | 0.0034 |
| RHC Only (n=139) | 371.7 | 11.02 | (10.21, 12.19) | 0.1841 | 1.00 | (0.94, 1.07) | 0.8790 |
| RHC&CHC (n=27) | 777.3 | 13.20 | (9.40, 17.00) | 0.1437 | 1.04 | (0.93, 1.16) | 0.5105 |
| Neither (n=349) | 477.8 | 10.35 | (9.57, 11.13) |  | referent | referent |  |
|  | Older adults (Ages 65+) | | | | | | |
| CHC Only (n=59) | 3860.0 | 63.68 | (58.12, 69.35) | 0.0003 | 0.84 | (0.81, 0.87) | <.0001 |
| RHC Only (n=138) | 606.2 | 76.79 | (72.69, 80.89) | 0.9959 | 0.96 | (0.94, 0.99) | 0.0025 |
| RHC&CHC (n=27) | 1337.9 | 78.19 | (58.06, 98.33) | 0.8898 | 0.88 | (0.84, 0.92) | <.0001 |
| Neither (n=343) | 837.7 | 76.79 | (72.69, 80.89) |  | referent | referent |  |
| **SOURCE:** Authors’ analysis using year 2002 State Inpatient Databases representing 8 states, and the 2002 Area Resource File; analysis for children limited to counties having at least 1,000 children ages 0-17; analysis of working age adults limited to counties having at least 1,000 adults ages 18-64; analysis of older adults limited to counties having at least 500 adults ages 65 or over.  **NOTES**: Adjusted analyses control for: physician supply; hospital bed supply; ED visit rates; hospitals with EDs, HMO penetration; rate of insurance; percent black, Hispanic, Asian, and American Indian; population change; education levels; population density; unemployment; death rates for heart disease, COPD, diabetes, and liver disease, and rural/urban location of county. | | | | | | | |
