# Supplementary material for: Association between community health center and rural health clinic presence and county-level hospitalization rates for ambulatory care sensitive conditions: an analysis across eight US states
Source: BMC Health Serv Res. 2009 Jul 31;9:134. doi: 10.1186/1472-6963-9-134 (PMC2727502; doi:10.1186/1472-6963-9-134)
Supplement: Additional file 2 — Factors influencing county population-level ACS hospitalization rates, by age group, eight states, 2002. [file 1472-6963-9-134-S2.doc]

Additional File 2. Factors influencing county population-level ACS hospitalization rates, by age group, eight states, 2002

|  | Children  (ages 0 – 17)  558 counties | | | Working age adults  (ages 18 – 64)  574 counties | | | Older adults  (ages 65+)  567 counties | | |
| --- | --- | --- | --- | --- | --- | --- | --- | --- | --- |
|  | *b* | SE | p-value | *b* | SE | p-value | *b* | SE | p-value |
| **Facilities (ref: neither)** |  |  |  |  |  |  |  |  |  |
| CHC Only | 0.0675 | 0.0684 | 0.3234 | -0.1473 | 0.0502 | 0.0034 | -0.1784 | 0.0183 | <.0001 |
| RHC Only | 0.0701 | 0.0468 | 0.1336 | 0.0049 | 0.0319 | 0.8790 | -0.0365 | 0.0121 | 0.0025 |
| RHC&CHC | 0.2657 | 0.088 | 0.0025 | 0.0372 | 0.0566 | 0.5105 | -0.1253 | 0.0230 | <.0001 |
| **Resources in county:** |  |  |  |  |  |  |  |  |  |
| MD/DO per 10,000 population | 0.0011 | 0.0023 | 0.6247 | 0.0011 | 0.0021 | 0.5859 | 0.0004 | 0.0008 | 0.5607 |
| Beds per 1,000 population | 0.0003 | 0.0072 | 0.9639 | -0.0106 | 0.0046 | 0.0200 | -0.0055 | 0.0016 | 0.0009 |
| Number of hospitals with ED | -0.0096 | 0.0143 | 0.4998 | -0.0104 | 0.0052 | 0.0467 | -0.0118 | 0.0021 | <.0001 |
| HMO penetration rate | 0.0004 | 0.0017 | 0.8168 | -0.0080 | 0.0012 | <.0001 | -0.0037 | 0.0004 | <.0001 |
| ED visits per 1000 population | 0.0008 | 0.0009 | 0.3477 | 0.0009 | 0.0006 | 0.1028 | 0.0012 | 0.0002 | <.0001 |
| Non-metropolitan county (v metro) | -0.0768 | 0.0578 | 0.1843 | -0.0456 | 0.0366 | 0.2129 | -0.0401 | 0.0134 | 0.0027 |
| **Characteristics of county population** |  |  |  |  |  |  |  |  |  |
| Percent population that is: |  |  |  |  |  |  |  |  |  |
| African American | -0.0003 | 0.0017 | 0.8587 | -0.0008 | 0.0010 | 0.3988 | -0.0014 | 0.0004 | 0.0001 |
| Hispanic white | -0.0043 | 0.0034 | 0.2123 | 0.0066 | 0.0013 | <.0001 | 0.0057 | 0.0005 | <.0001 |
| Asian | 0.0288 | 0.0205 | 0.1597 | -0.0012 | 0.0025 | 0.6251 | 0.0018 | 0.0009 | 0.0362 |
| American Indian / Native  American | -0.0202 | 0.0097 | 0.0376 | 0.0024 | 0.0013 | 0.0608 | 0.0002 | 0.0005 | 0.6432 |
| Population change, 1990 - 2000, % |  |  |  | -0.0161 | 0.0013 | <.0001 | -0.0117 | 0.0004 | <.0001 |
| Percent with less than high school education | -0.0008 | 0.0022 | 0.7031 | 0.0019 | 0.0024 | 0.4251 | 0.0026 | 0.0009 | 0.0039 |
| Population per square mile (/100) | 0.0336 | 0.0035 | <.0001 | 0.0011 | 0.0022 | 0.6123 | 0.0008 | 0.0008 | 0.3075 |
| Percent unemployed (/10) | -0.0015 | 0.001 | 0.1325 | 0.0551 | 0.0495 | 0.2659 | 0.1099 | 0.0185 | <.0001 |
| Percent uninsured | -0.1481 | 0.0694 | 0.0329 | 0.0429 | 0.0028 | <.0001 | 0.0271 | 0.0011 | <.0001 |
| Median household income (thousands) | -0.0264 | 0.0081 | 0.0012 | 0.0069 | 0.0023 | 0.0023 | 0.0050 | 0.0009 | <.0001 |
| **Death rates (x10,000) for:** | -0.0044 | 0.0053 | 0.4017 |  |  |  |  |  |  |
| Cardiovascular disease |  |  |  | 0.0165 | 0.0024 | <.0001 | 0.0105 | 0.0009 | <.0001 |
| Chronic obstructive pulmonary disease | -0.0079 | 0.0037 | 0.0335 | 0.0088 | 0.0083 | 0.2878 | 0.0185 | 0.0031 | <.0001 |
| Diabetes | 0.0117 | 0.0133 | 0.3770 | -0.0346 | 0.0118 | 0.0033 | -0.0225 | 0.0043 | <.0001 |
| Liver disease | -0.0326 | 0.0194 | 0.0932 | -0.0186 | 0.0227 | 0.4128 | -0.0252 | 0.0085 | 0.0029 |
| **Source:**  Authors’ analysis using year 2002 State Inpatient Databases representing 8 states, and the 2002 Area Resource File; death rates based on 3-year average; estimates from Poisson regression analysis; *b*=model coefficient. | | | | | | | | | |
